# Supplementary material for: Comparing Gleason Pattern 4 Measurement Approaches on Prostate Biopsy Using Machine Learning: A Proof-of-Principle Study
Source: medRxiv. 2026 Apr 24:2026.04.23.26351615. Preprint. [Version 1] doi: 10.64898/2026.04.23.26351615 (PMC13131693; doi:10.64898/2026.04.23.26351615)

medRxiv preprint doi: https://doi.org/10.64898/2026.04.23.26351615; this version posted April 24, 2026. The copyright holder has placed this preprint (which was not certified by peer review) in the Public Domain. It is no longer restricted by copyright. Anyone can legally share, reuse, remix, or adapt this material for any purpose without crediting the original authors.

Supplementary Table 1. Pattern 4 lengths (mm) by proportion of either cancer or tissue for each of the 15 measurement types by biopsy Gleason Grade. Values are expressed as median (IQR).

**Characteristic**	**GG2** N = 486	**GG3** N = 183	**GG4** N = 57
All foci by cancer	0.35 (0.12, 0.98)	4.71 (2.67, 8.03)	8.61 (4.96, 13.69)
All foci by tissue	0.30 (0.10, 0.80)	3.90 (1.98, 6.75)	7.54 (3.52, 11.99)
All foci by pixel-counting	0.30 (0.09, 0.80)	4.42 (2.01, 7.52)	7.83 (4.14, 14.04)
Largest focus by cancer	0.27 (0.09, 0.68)	3.25 (1.58, 5.76)	6.57 (3.20, 10.68)
Largest focus by tissue	0.23 (0.07, 0.62)	3.03 (1.28, 5.10)	6.04 (3.00, 10.09)
Interfocal stroma included when the distance between the two is:			
Less than 3mm (cancer denominator)	0.48 (0.18, 1.24)	6.39 (3.84, 10.23)	11.59 (8.29, 16.78)
Less than 3mm (tissue denominator)	0.31 (0.10, 0.81)	4.07 (2.04, 6.84)	7.74 (3.63, 12.40)
Less than 1mm (cancer denominator)	0.39 (0.14, 1.10)	5.48 (3.07, 8.93)	9.36 (6.23, 14.23)
Less than 1mm (tissue denominator)	0.30 (0.10, 0.81)	3.80 (2.00, 6.80)	7.74 (3.66, 12.09)
Smaller than the combined lengths of the two adjacent foci (cancer denominator)	0.42 (0.14, 1.09)	5.47 (3.18, 9.32)	10.71 (6.66, 15.43)
Smaller than the combined lengths of the two adjacent foci (tissue denominator)	0.30 (0.10, 0.81)	4.04 (2.00, 6.84)	7.74 (3.66, 12.38)
Smaller than twice the combined lengths of the two adjacent foci (cancer denominator)	0.45 (0.16, 1.26)	6.04 (3.65, 10.11)	11.40 (7.03, 16.23)
Smaller than twice the combined lengths of the two adjacent foci (tissue denominator)	0.30 (0.10, 0.81)	4.07 (2.03, 6.85)	7.74 (3.76, 12.40)
Smaller than the shorter of the two adjacent foci (cancer denominator)	0.37 (0.12, 1.04)	4.79 (2.75, 8.67)	8.75 (5.38, 13.80)
Smaller than the shorter of the two adjacent foci (tissue denominator)	0.30 (0.10, 0.80)	3.90 (1.99, 6.76)	7.62 (3.52, 12.02)

medRxiv preprint doi: https://doi.org/10.64898/2026.04.23.26351615; this version posted April 24, 2026. The copyright holder has placed this preprint (which was not certified by peer review) in the Public Domain. It is no longer restricted by copyright. Anyone can legally share, reuse, remix, or adapt this material for any purpose without crediting the original authors.

Supplementary Table 2. Discrimination (AUC) of the alternative definition of advanced disease where EPE is also considered advanced disease by each of the 15 measurement types.

**Measurement**	**AUC**	**95% CI**		
Grade group (pathology report)	0.577	0.540, 0.614		
Grade group (ML)	0.536	0.502, 0.571		
Percentage pattern 4	0.559	0.517, 0.601		
Pixel counting (area) method	0.612	0.571, 0.653		
	**Cancer as the denominator**		**Tissue as the denominator**	
All foci	0.592	0.550, 0.633	0.596	0.555, 0.637
Largest focus	0.589	0.547, 0.630	0.591	0.549, 0.632
**Interfocal stroma included with rule:**				
Less than 3mm	0.588	0.547, 0.629	0.595	0.554, 0.636
Less than 1mm	0.590	0.548, 0.631	0.596	0.555, 0.637
Smaller than the combined lengths of the two adjacent foci	0.588	0.547, 0.630	0.595	0.554, 0.637
Smaller than twice the combined lengths of the two adjacent foci	0.585	0.543, 0.626	0.595	0.554, 0.636
Smaller than the shorter of the two adjacent foci	0.591	0.550, 0.632	0.596	0.555, 0.637

medRxiv preprint doi: https://doi.org/10.64898/2026.04.23.26351615; this version posted April 24, 2026. The copyright holder has placed this preprint (which was not certified by peer review) in the Public Domain. It is no longer restricted by copyright. Anyone can legally share, reuse, remix, or adapt this material for any purpose without crediting the original authors.

Supplementary methods

Digitized prostate biopsy slides were analyzed using a machine-learning algorithm (PAIGE-AI) to quantify tissue area, cancer area, and Gleason pattern 4 (GP4). Size-thresholding was applied to exclude isolated regions smaller than 700 μm^2. Each biopsy core may be sectioned at multiple histologic levels; these levels appear on a slide as separate objects, all corresponding to the same physical biopsy core.

Object-level measurements, including core length and GP4 length, were generated for each level. To exclude tissue fragments and artifacts, object-level core lengths within each slide were reviewed; small objects that deviated substantially from the dominant core length were classified as fragments and removed. Slides with extensive fragmentation, artifacts, or no measurable GP4 were excluded.

For each biopsy core, GP4 length was calculated as the average GP4 length across its sectioned levels. Patient-level GP4 burden was then computed by summing the average GP4 length across all included biopsy cores for that patient.

To illustrate the fifteen different GP4 quantification approaches, we use a single biopsy core example below, where light blue is normal tissue, dark blue is pattern 3 and dark green is pattern 4. Three cancer foci are separated by interfocal stroma measuring 0.9 mm and 3 mm. The total cancer length is 7 mm, of which 5 mm is GP4. Each focus occupies approximately 50% of the core width.

We first distinguish between foci inclusion, where we either sum measurements across all foci or measure only the longest focus. In this case, the largest focus is 3 mm long, 2 mm of which is GP4 to give a 67% GP4 percentage. Using a cancer-based denominator, the GP4 fraction is 3 × 0.67, yielding 2.0 mm of GP4. The focus spans half the core such as that GP4 is 33% of the tissue, hence using a tissue as the denominator give 3 × 0.33 = 1.0 mm of GP4.

The next question is whether the interfocal stroma would be included under different inclusion rules. Interfocal stroma was included based on five rules: including interfocal stroma < 3 mm; 1 mm; shorter than the combined lengths of adjacent foci; shorter than twice the combined lengths; or shorter than the shorter adjacent focus.

Under these rules, the 0.9 mm interfocal stroma would be included by all five rules (<3 mm, <1 mm, shorter than the combined lengths of adjacent foci, shorter than twice the combined lengths, and shorter than the shorter adjacent focus). The 3 mm interfocal stroma would only be included by the two relative-length rules based on the combined lengths of the adjacent foci (shorter than the combined lengths and shorter than twice the

medRxiv preprint doi: https://doi.org/10.64898/2026.04.23.26351615; this version posted April 24, 2026. The copyright holder has placed this preprint (which was not certified by peer review) in the Public Domain. It is no longer restricted by copyright. Anyone can legally share, reuse, remix, or adapt this material for any purpose without crediting the original authors.

combined lengths). Now that interfocal stroma is included under some rules, we next consider whether cancer or tissue is used as the denominator.

If cancer is used as the denominator, the calculation is as follows: the total cancer length is 7 mm. When the 0.9 mm interfocal stroma is included, the counted length becomes 7.9 mm. The fraction of cancer that is GP4 is 5 ÷ 7. Multiplying the counted length by this fraction yields 5.6 mm of GP4. If both interfocal stromal segments are included, the counted length becomes 10.9 mm and multiplying by 5 ÷ 7 yields 7.8 mm of GP4.

If tissue is used as the denominator, the calculation changes. Ignoring interfocal stroma, the total tissue length is 7 mm. Of this tissue, 46.7% is GP4, calculated as (2mm × 0.5 + 1.5 mm × 1.0 + 1.5 mm × 0.5)/7 mm, yielding 3.25 mm of GP4. Including the 1 mm interfocal stroma increases the total tissue length to 8 mm, reducing the GP4 fraction to 40.6%, but the product remains 3.25 mm. Including both interfocal stromal segments increases the tissue length to 11 mm and reduces the GP4 fraction to 29.5%, again yielding mm of GP4. In other words, when tissue is used as the denominator, explicitly calculating interfocal stroma does not change the GP4 estimate. Including interfocal stroma increases the counted length, but this is exactly offset by the corresponding decrease in the GP4 fraction, resulting in the same final value.

Finally, pixel areas were converted to microns using slide-specific microns-per-pixel (mpp) values, and the resulting GP4 area was converted to a linear extent by dividing by the average core width. This is equivalent to estimating the percentage of the core occupied by GP4 and multiplying by the total core length. This method considers variations in the width of a core over its length. If the width of the GP4 at each focus is the same as the average core width, the length calculated by this method will match the all-foci tissue-based approach. See Supplementary Figure 1 and 2.

medRxiv preprint doi: https://doi.org/10.64898/2026.04.23.26351615; this version posted April 24, 2026. The copyright holder has placed this preprint (which was not certified by peer review) in the Public Domain. It is no longer restricted by copyright. Anyone can legally share, reuse, remix, or adapt this material for any purpose without crediting the original authors.

Methodologic challenges for ML measurement of GP4

1. The algorithm was designed to measure lengths by drawing a consistent end-to-end midline along each core. We noticed that in some cases, small tissue irregularities would cause the midline to deviate, leading to inaccurate measurements. Example shown in Supplementary Figure 3.

2. The algorithm quantified object lengths in pixels and converted them to microns using a predefined scale factor. However, we observed substantial discrepancies in the measured specimen lengths. Further investigation revealed that these inconsistencies were due to variations in slide magnification. To address this, we adjusted the pixel-to-micron conversion rate to account for magnification differences.

3. During the initial phases of the project, some fragments were recorded as having a total length of zero. Further investigation revealed that when a fragment of a biopsy core contained no GP4, the algorithm assigned a total length of zero to that entire object. This behavior resulted in an underestimation of the total tissue length for the core. For example, in Supplementary figure 4 we have 2 fragments of a biopsy core where the first fragment would have been considered a length of zero, thus underestimating the total tissue length by half.

4. We found large discrepancies in GP4 lengths across levels from the same core when interfocal stroma was included. After manual review, we observed that in several cases, the algorithm labeled scattered pixels of GP4 on a fragment, which distorted the measurements. To prevent these small detections from affecting the results, we implemented a minimum threshold for GP4 foci before they were included in the length calculations. Example shown in Supplementary Figure 5.

5. During histopathologic processing, biopsy fragments are sectioned at multiple levels to enable evaluation of the same core at varying depths. As a result, multiple representations of the same biopsy core may appear on a single slide. The machine learning model had difficulty distinguishing different levels of the same core from distinct cores present on the slide. For this, manual review was required to verify core identity and ensure accurate measurements. Example shown in Supplementary Figure 6.

medRxiv preprint doi: https://doi.org/10.64898/2026.04.23.26351615; this version posted April 24, 2026. The copyright holder has placed this preprint (which was not certified by peer review) in the Public Domain. It is no longer restricted by copyright. Anyone can legally share, reuse, remix, or adapt this material for any purpose without crediting the original authors.

6. In some cases, multiple biopsy fragments were present within a single biopsy core, which inflated the measured tissue or cancer size. Manual review was necessary to identify these instances and adjust the measurements by accounting for the number of fragments present. Example shown in Supplementary Figure 7.

8. Due to storage-related issues, the machine learning system occasionally saved biopsy slides containing multiple levels as separate images. As a result, these images were incorrectly treated as distinct biopsy fragments. Manual review was required to identify such cases and exclude duplicate measurements.

9. During histopathological processing, biopsy fragments may become fragmented, resulting in discontinuous foci. In such cases, manual review was required to determine whether fragmentation was so severe it would exclude the respective core from the analysis. Example shown in Supplementary Figure 8.

medRxiv preprint doi: https://doi.org/10.64898/2026.04.23.26351615; this version posted April 24, 2026. The copyright holder has placed this preprint (which was not certified by peer review) in the Public Domain. It is no longer restricted by copyright. Anyone can legally share, reuse, remix, or adapt this material for any purpose without crediting the original authors.

Supplementary Figure 1. Scheme of a hypothetical biopsy core. Light blue: benign tissue. Dark Blue: Gleason pattern 3. Green: Gleason pattern 4. All values are given in mm.

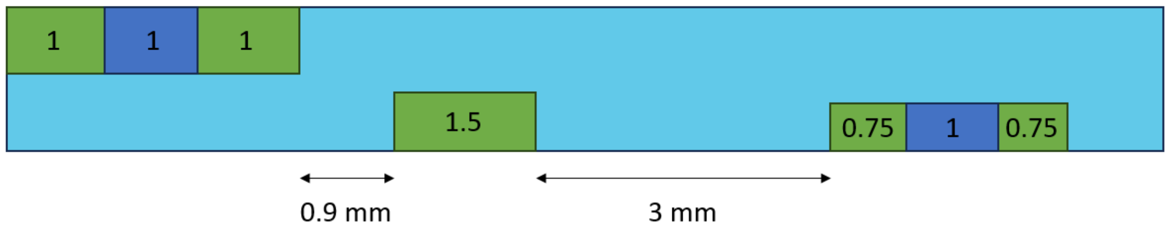

medRxiv preprint doi: https://doi.org/10.64898/2026.04.23.26351615; this version posted April 24, 2026. The copyright holder has placed this preprint (which was not certified by peer review) in the Public Domain. It is no longer restricted by copyright. Anyone can legally share, reuse, remix, or adapt this material for any purpose without crediting the original authors.

Supplementary Figure 2. Left panel: Hematoxylin-eosin-stained biopsy slide from a patient with prostate cancer. Right panel: Digitized image of the corresponding biopsy slide. Light blue indicates benign tissue, dark blue indicates Gleason Pattern 3 (GP3), and dark green indicates Gleason Pattern 4 (GP4). The measured length (microns) of the cancer focus is highlighted and annotated in red and the measured length of interfocal stroma is annotated in green.

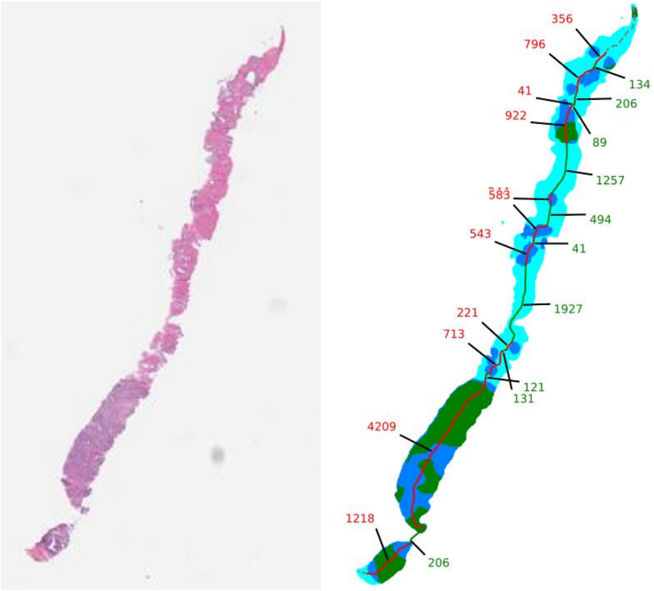

medRxiv preprint doi: https://doi.org/10.64898/2026.04.23.26351615; this version posted April 24, 2026. The copyright holder has placed this preprint (which was not certified by peer review) in the Public Domain. It is no longer restricted by copyright. Anyone can legally share, reuse, remix, or adapt this material for any purpose without crediting the original authors.

Supplementary Figure 3. Example of biopsy slide with midline deviation.

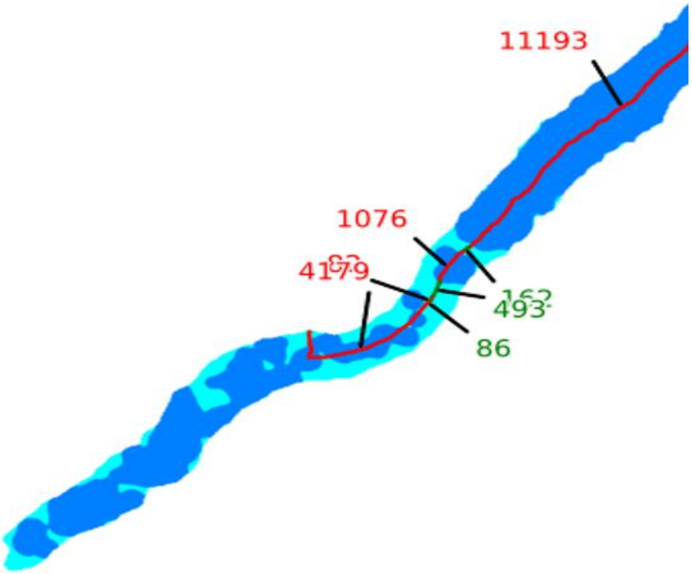

medRxiv preprint doi: https://doi.org/10.64898/2026.04.23.26351615; this version posted April 24, 2026. The copyright holder has placed this preprint (which was not certified by peer review) in the Public Domain. It is no longer restricted by copyright. Anyone can legally share, reuse, remix, or adapt this material for any purpose without crediting the original authors.

Supplementary Figure 4. Example of biopsy slide with no pattern 4 on one core.

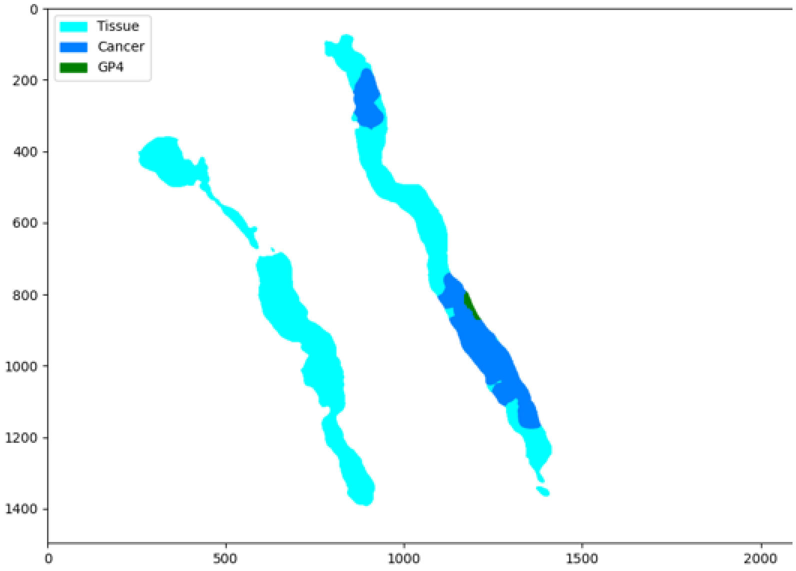

medRxiv preprint doi: https://doi.org/10.64898/2026.04.23.26351615; this version posted April 24, 2026. The copyright holder has placed this preprint (which was not certified by peer review) in the Public Domain. It is no longer restricted by copyright. Anyone can legally share, reuse, remix, or adapt this material for any purpose without crediting the original authors.

Supplementary Figure 5. Example of biopsy slide with scattered pixels of pattern 4.

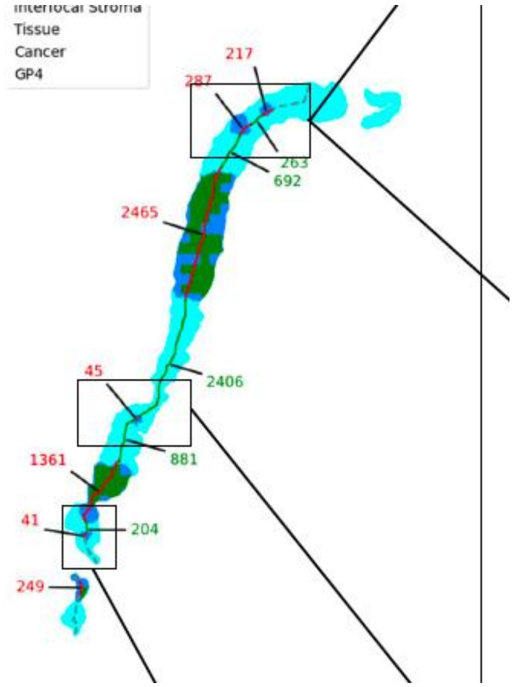

medRxiv preprint doi: https://doi.org/10.64898/2026.04.23.26351615; this version posted April 24, 2026. The copyright holder has placed this preprint (which was not certified by peer review) in the Public Domain. It is no longer restricted by copyright. Anyone can legally share, reuse, remix, or adapt this material for any purpose without crediting the original authors.

Supplementary Figure 6. Example of biopsy slide with multiple different levels of the same core.

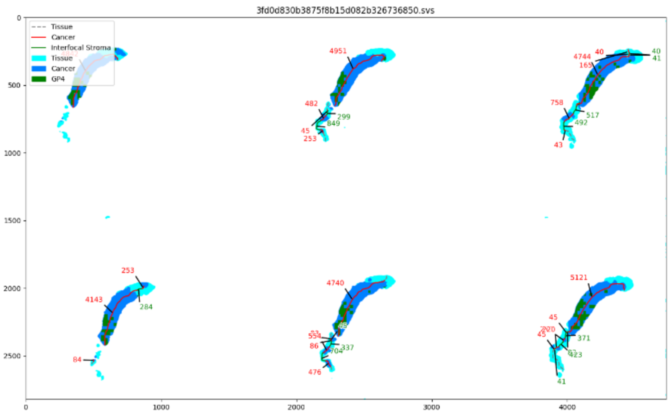

medRxiv preprint doi: https://doi.org/10.64898/2026.04.23.26351615; this version posted April 24, 2026. The copyright holder has placed this preprint (which was not certified by peer review) in the Public Domain. It is no longer restricted by copyright. Anyone can legally share, reuse, remix, or adapt this material for any purpose without crediting the original authors.

Supplementary Figure 7. Example of biopsy slide with multiple cores and multiple levels

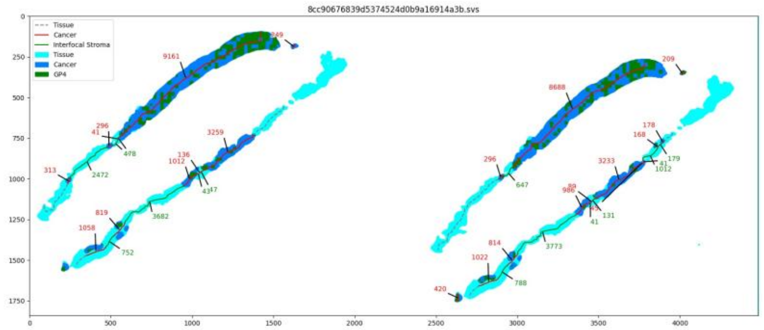

medRxiv preprint doi: https://doi.org/10.64898/2026.04.23.26351615; this version posted April 24, 2026. The copyright holder has placed this preprint (which was not certified by peer review) in the Public Domain. It is no longer restricted by copyright. Anyone can legally share, reuse, remix, or adapt this material for any purpose without crediting the original authors.

Supplementary Figure 8. Example of a completely fragmented biopsy core that required exclusion from the analysis.

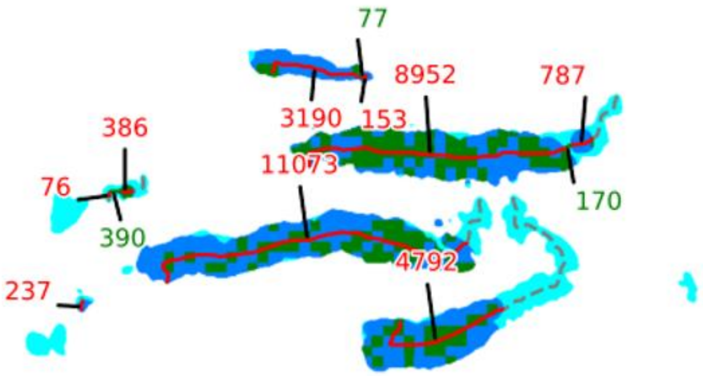

Supplement: 1 [file NIHPP2026.04.23.26351615v1-supplement-1.pdf]
